# Effects of vitamin D3 supplementation on clinical symptoms, quality of life, serum serotonin (5-hydroxytryptamine), 5-hydroxy-indole acetic acid, and ratio of 5-HIAA/5-HT in patients with diarrhea-predominant irritable bowel syndrome: A randomized clinical trial

**DOI:** 10.17179/excli2020-2247

**Published:** 2020-05-19

**Authors:** Masoumeh Khalighi Sikaroudi, Marjan Mokhtare, Farzad Shidfar, Leila Janani, AmirHossein Faghihi Kashani, Mohsen Masoodi, Shahram Agah, Afsaneh Dehnad, Shahrzad Shidfar

**Affiliations:** 1 Department of Nutrition, School of Public Health, Iran University of Medical Sciences, Tehran, Iran; 2 Colorectal Research Center, Rasoul-e-Akram Hospital, Iran University of Medical Sciences, Tehran, Iran; 3 Department of Biostatistics, School of Public Health, Iran University of Medical Sciences, Tehran, Iran; 4 Department of English Language, School of Health Management and Information Sciences, Iran University of Medical Sciences, Tehran, Iran; 5 Worcester Memorial Hospital, University of Massachusetts, Worcester, U.S.A.

**Keywords:** irritable bowel syndrome, vitamin D3, clinical symptoms, serotonin, randomized controlled trial

## Abstract

Vitamin D deficiency, common in the population with irritable bowel syndrome (IBS), can induce the main factors that lead to IBS clinical symptoms, such as depression, anxiety, and inflammation. Serotonin (5-HT) plays an important role in the pathophysiology of IBS, and its production and secretion are increased from the lumen due to stress and inflammation. The aim of this study was to evaluate the effect of vitamin D3 supplementation on the pathogenesis of diarrhea-predominant IBS (IBS-D). Seventy-four IBS-D patients (age: 18-65 y) participated in a randomized, double-blind, placebo-controlled trial study from February 2017 to May 2018, at Rasoul-e-Akram Hospital, Tehran, Iran. Subjects were allocated into two groups receiving 50,000 IU/week of vitamin D3 or placebo for 9 weeks. IBS severity score system (IBS-SSS), IBS-quality of life questionnaire (QoL), hospital anxiety and depression Scale (HADs), visceral sensitivity index (VSI) and serum 25(OH) vitamin D3, serotonin, 5-hydroxy-indole acetic acid and ratio of 5-HIAA/5-HT were evaluated before and after the interventions. Symptoms severity, QoL, HADs-depression, and VSI score improved significantly in the vitamin D group as compared to the placebo group (P-values: <0.001, 0.049, 0.023, and 0.008; respectively). There were no significant differences in abdominal bloating, HADs-anxiety, serum 5-HT, 5-HIAA, and 5-HIAA/5-HT between the two groups at the end of the study. Based on our results, we recommend serum vitamin D be evaluated in the process of treatment of these patients to ameliorate symptoms and quality life of IBS-D patients with vitamin D deficiency and/or insufficiency.

## Abbreviations

IBS: Irritable Bowel Syndrome

IBS-D: diarrhea-predominant IBS

IBS-C: constipation-predominant IBS

IBS-SSS: IBS severity score system

IBS-QoL: IBS-quality of life questionnaire

HADs: Hospital Anxiety and Depression Scale

VSI: Visceral sensitivity index

5-HT: 5-hydroxytryptamine

5-HIAA: 5-hydroxy-indole acetic acid

TPH 1,2: tryptophan hydroxylase 1,2

SERT: serotonin reuptake transporter

## Introduction

Irritable bowel syndrome (IBS) is a chronic functional gastrointestinal disorder characterized by abdominal pain associated with altered bowel habits (Mearin et al., 2016[44]). Diagnosis of IBS is recognized by ROME IV criteria that is based on clinical symptoms (Drossman, 2016[11]; Ford et al., 2014[18]). Epidemiological studies have shown that IBS is more common in women and young people aged (25-54 years old) (Lacy and Moreau, 2016[35]), and its prevalence is 5 %-20 % (Ford and Talley 2012[19]). According to the bowel habits, IBS is classified to diarrhea-predominant IBS (IBS-D), constipation-predominant IBS (IBS-C), and mixed-type IBS (IBS-M) which has alternating periods of diarrhea and constipation (Ford et al., 2014[18]). 

IBS is a multifactorial disorder with the causes which are not still well understood. Genetic, gastrointestinal disorders, anxiety, disturbances in brain-gut axis, infection and inflammation in bowel, and changes in intestinal microbiota can be associated with IBS symptoms (Lacy and Moreau, 2016[35]; Nanayakkara et al., 2016[46]; Saha, 2014[58]). According to the psychological studies and American Association of Gastroenterology (AGA), depression and anxiety, which have been observed in 60 % of IBS patients, increase gastrointestinal symptoms including the severity of diarrhea and abdominal discomfort (Drossman et al., 2002[12]; Levy et al., 2006[38]). Anxiety is often the main cause of visceral pain hypersensitivity in patients with IBS (Labus et al., 2004[33]). IBS has a strong impact on quality of life such as diet, physical appearance, work, education, and interpersonal relationships (Phillips et al., 2013[54]).

Serotonin or 5-hydroxy tryptamine (5-HT) is a key neurotransmitter of the enteric nervous system (ENS) playing a significant role in the control of gastrointestinal motility, sensation, and secretion (Sen et al., 2011[60]). It seems that serotonin has a significant role in the pathophysiology of the IBS (Garvin and Wiley, 2008[22]). Approximately 95 % of total body 5-HT is found in the GI tract, 90 % of which is produced by enterochromaffin cells and 10 % by serotonergic neurons of the myenteric plexus (Sen et al., 2011[60]). Blood serotonin levels are often increased in IBS-D and decreased in IBS-C (Spiller et al., 2007[62]). The abnormal blood serotonin levels in patients with IBS is because all the 5-HT, found in the blood, is derived from the GI tract (Atkinson et al., 2006[4]). On the other hand, the number of enterochromaffin cells in the colon of patients with IBS-D is higher than that of healthy people, and even in patients with ulcerative colitis (Gershon et al., 1965[23]; Sen et al., 2011[60]). Upon the uptake of 5-HT into enterocyte and pre-synaptic neurons by the Serotonin Reuptake Transporter (SERT in the lumen), it is broken down to 5-hydroxy indole acetic acid (5-HIAA) by intracellular monoamine oxidase (MAO). Blood 5-HT is converted to 5-HIAA by MAO and aldehyde dehydrogenase in the liver and kidney and is then excreted in the urine (Thijssen et al., 2016[65]; Yazar et al., 2005[68]). The number of SERTs, their activities and the production of 5-HIAA are reported to be decreased in patients with IBS-D (Thijssen et al., 2016[65]). In addition, animal studies have shown that nonspecific inflammation can cause EC cells hyperplasia and decrease expression of SERT (Linden et al., 2003[41]), leading to an increase in 5-HT at the mucosal level and followed by the incremental gut motility and secretion, thereby causing IBS symptoms (Dunlop et al., 2005[13]).

1,25-dihydroxy vitamin D3 [1,25(OH)_2_D3] is a physiologically active form of vitamin D3 the main role of which is modulation of cell growth, immune function and reduction of inflammation (National Institutes of Health, Office of Dietary Supplements - Vitamin D, 2020[47]). It has been estimated that 1 billion people worldwide have vitamin D deficiency or insufficiency (Holick 2007[24]). Studies have shown that in patients with IBS, vitamin D deficiency is more common than in healthy population and severity of symptoms is inversely correlated with serum vitamin D concentration (Williams et al., 2018[67]). However, there are a few studies about the effect of vitamin D supplementation on IBS symptoms (Abbasnezhad et al., 2016[1]; El Amrousy et al., 2018[15]; Jalili et al., 2016[27]; Sprake et al., 2012[63]; Tazzyman et al., 2015[64]). Three recent randomized clinical trial studies have reported that treatment with vitamin D in all classification of IBS patients who had vitamin D deficiency could relief symptoms and quality of life compared to the placebo group (Abbasnezhad et al., 2016[1]; El Amrousy et al., 2018[15]; Jalili et al., 2016[27]). However, the mechanism of this effect is unclear. There are some hypotheses that vitamin D may improve clinical symptoms in patients with IBS by ameliorating anxiety and depression (Parker et al., 2017[49]), anti-inflammatory effects (Li et al., 2015[40]; Mangin et al., 2014[43]), and regulating the composition of the gut-microbiota and gut barrier function (Cantorna et al., 2014[8]; Luthold et al., 2017[42]). 

To the best of our knowledge the present study is the first one assessing the effects of the increase in serum vitamin D on different dimensions of irritable bowel syndrome. The aim of this study was to evaluate the effect of vitamin D3 supplementation on improving clinical outcomes, quality of life, and anxiety, and decreasing serum serotonin (5-hydroxytryptamine), increasing 5-hydroxy-indole acetic acid and also 5-HIAA/5-HT ratio in patients with diarrhea-predominant irritable bowel syndrome.

## Methods

### Study design and participants

This was a randomized, double-blind, placebo-controlled trial study with parallel design performed in 9 weeks. The participants were informed about the study via printed posters on the notice boards of Rasoul-e-Akram Hospital, Tehran, Iran. We also explained the study objectives verbally to the patients with IBS-D, who were referred to Gastrointestinal Clinic of the hospital. Eventually, we recruited 88 adult male and female volunteers, with IBS-D, between the ages of 18- 65 years old. ROME IV criteria (Drossman, 2016[11]) and World Gastroenterology Organization (WGO) questionnaire, for healthcare professional (HCP) of IBS patients (Quigley et al., 2016[55]), were used as diagnostic criteria. The study was carried out from February 2017 to May 2018. The inclusion criteria for including the patients were: having irritable bowel syndrome with diarrhea-predominant, IBS-SSS score between 175 to 300, not being pregnant or lactating, having no GI disorders such as inflammatory bowel disease, celiac, GI infection or history of colon cancer, intestinal surgery or radiotherapy, and cholecystectomy, not taking vitamin D supplement in the last 6 months, no use of other supplements, NSIADs, Glucocorticoid and antidepressants drug containing serotonin resorptive antagonists, selective serotonin reuptake inhibitors (SSRIs), tricyclic antidepressants, no alcohol and caffeine intake and also no smoking 12 hours before the test. Exclusion criteria were: serum vitamin D higher than 30 ng/ml, any abnormal response or side effect to supplementation, blood in the stool, fast weight lost, using lower than 80 % of supplements, reluctance to continue cooperation with the researchers. Demographic data, medical history, measuring anthropometric indicators, physical activity [measured by International Physical Activity Questionnaire (IPAQ)], and 3-day food diary records (2 working day and 1 holiday) [their nutrients were calculated by dietary calculator software Nutritionist IV] were obtained from participants at baseline and the end of the study.

### Ethics statements

The study was approved by ethic committee of Iran University of Medical Sciences (IR.IUMS.REC 1395.9413323001) and the study protocol was registered in the Iranian clinical trials Web site at: (http://www.irct.ir: IRCT201701162709N42). The objective of the study was explicitly stated to the participants and written informed consent form for acceptance of study details was signed by all participants.

### Intervention

Following the randomization, participants received weekly Vitamin D3 (50,000 IU) or placebos (filled by edible paraffin) pearls (Zahravi Company, Tabriz, Iran) for 9 weeks. Placebos were similar to vitamin D in shape, color and package. Patients received 8 pearls weekly for 8 weeks and the last pearl used in 10^th^ week of intervention. According to protocol treatment of vitamin D deficiency, the serum level of vitamin D can be improved by intake of 50,000 IU vitamin D3 in 8 weeks and after that using only 1 pearl monthly for maintaining serum vitamin D level in a normal range will suffice (Roth et al., 2012[56]) as 10-14 days after using the pearls, vitamin D reaches to peak rate in serum (Jones 2008[29]). For this reason, the blood sampling from participants were performed in week 12. In addition, all patients received Mebeverine 135 mg twice a day beside the supplementation.

### Outcomes

The primary outcome was assessment of IBS severity score system. Secondary outcomes were assessment of quality of life, stress and depression, visceral sensitivity, serum serotonin and 5-hydroxyindole acetic acid. 

### Sample size calculation

According to our primary outcome and based on the result of similar previous study by Abbasnezhad et al. (2016[1]) the sample size was calculated to be 44 subjects in each group by considering 22 unit change in the average of IBS severity score system with a type I error of 5 % (α = 0.05), a type II error of 20 % (β = 0.2; power = 80 %), and considering 20 % of loss to follow up. We used G-Power software (http://www.gpower.hhu.de) for calculation of the sample size.

### Randomization and allocation 

The quadruple block randomization was used for allocation of the participants. According to the sample size of 88, 22 blocks were produced by using the online site (http://www.sealedenvelope.com). For making the concealment in the randomization process, dedicated codes were produced by the software and were used on the pharmaceutical sheets.

### Information gathering tools

#### Gastrointestinal symptoms and severity scale

IBS symptoms severity score system (IBS-SSS) questionnaire (Francis et al., 1997[20]) was used for monitoring the IBS symptoms and severity at the baseline and end of the study. Data were collected by interview using visual analog scale (VAS). This questionnaire contains 5 items describing the severity of symptoms within the previous 10 days, such as: abdominal pain severity, abdominal pain duration, abdominal distention severity, bowel habit satisfaction, and life disruption. Each item was scored from 0 to 100 (total score from 0 to 500). The obtained scores were assigned “mild”, “moderate” and “severe” scores by scoring from 75 to 175, 175 to 300, and >300. Patients scoring below 75 were considered to be in remission.

### Quality of life

IBS-QoL questionnaire (Patrick et al., 1998[50]) consists of 34 items and 8 subscales (dysphoria, interference with activity, body image, health worry, food avoidance, social reaction, sexual desire, and relationships) were filled for the participants. Each item has a 5-point response scale (1= not at all to 5= extremely). Similar to a previous study (Mokhtare et al., 2018[45]), we used the raw total score of the questionnaire with the range of 34 to 170, indicating that higher scores demonstrated lower quality of life in individuals.

#### Hospital Anxiety and Depression scale (HADs)

We used HADs (Larsen et al., 2014[36]; Zigmond and Snaith 1983[69]) questionnaire, which is a 14-item self-rating scale, assessing anxiety and depression symptoms in different diseases associated with psychological disorders. The questionnaire has two parts consisting of seven questions related to anxiety (HADs-ANX), and seven to depression (HADs-DEP). Each item has a rating of 0 to 3; the higher rates indicate greater anxiety or depression symptoms. The total score was calculated by the sum of the concession items, and ranged in each part from 0 to 21. The scores from 0 to 7 indicated a normal scale, 8 to 10 borderline, and the scores between 11 to 21 illustrated clinical problems.

#### Visceral sensitivity index (VSI)

We also used VSI questionnaire (Labus et al., 2004[33]) to evaluate GI symptom-specific anxiety (GSA) in IBS patients. It has a 6-point Likert scale for each item (0= strongly disagree to 5= strongly agree) and total score ranges from 0 to 75, which a higher score indicates greater GSA. The validity and reliability of the VSI have been confirmed in several studies (Labus et al., 2007[34], 2004[33]; Saigo et al., 2014[59]); however, this questionnaire has not been evaluated in Iran. Thus, we designed the test-retest experiment with 2 weeks interval between two tests in 20 IBS-D patients. The original VSI questionnaire was translated into Persian by a fluent translator, and then a native English speaker back-translated it into English. Afterwards, four gastroenterologist experts confirmed the content of the questionnaire. At the end, Cronbach’s α was estimated 0.914 for reliability of VSI questionnaire in Persian. 

#### Laboratory test

Following an overnight fasting, 5 ml blood samples were taken from the participants 90 minutes after ingestion of a carbohydrate-rich breakfast with 450 Kcal at the baseline and end of the study. Based on previous studies that evaluated 5-HT and 5-HIAA in IBS patients (Bearcroft et al., 1998[6]; Gershon et al., 1965[23]; Houghton et al., 2003[26]; Park et al., 2009[48]), subjects were given a breakfast containing 60 gr white bread, 35 gr cheese, 5 gr butter, 3 tablespoons of carrot jam and 150 ml water within 10 minutes.

Blood samples were collected in the clot activator tubes and serum samples were left at room temperature and then centrifuged twice at 4,000 rpm for 10 minutes for each centrifuge in order to, ensure no platelet contamination of supernatant in the samples because 5-HT is rapidly taken up by platelets which contain the largest 5-HT in the peripheral blood (Da Prada et al., 1972[10]). Serum samples were stored at -80 °C for later lab analysis.

##### Vitamin D

We used the LIAISON® 25 (OH) Vitamin D3 assay (DiaSorin, USA) which is a direct competitive chemiluminescence immunoassay (CLIA) for quantitative determination of total 25 (OH) vitamin D3 in serum. Functional sensitivity from the regression equation of dose concentration of 25 (OH) Vitamin D3 is ≤4.0 ng/mL. The classification of 25 (OH) Vitamin D3 status is <10 ng/mL deficiency, 10-30 ng/mL insufficiency, 30-100 ng/mL sufficiency, and >100 ng/mL toxicity (National Institutes of Health, Office of Dietary Supplements - Vitamin D, 2020[47]).

##### Serotonin (5-Hydroxy Tryptamine)

We used the ELISA method by IBL kit package (Hamburg, Germany) for the quantitative determination of Serotonin in human serum. Due to the dilution of samples, the values acquired from tests had to be multiplied by 107 to obtain the serotonin concentrations in ng/ml. To convert the serotonin unit from ng/ml to nmol/l, we multiplied the obtained value by 5.7. The intra-assay coefficient of variability (CV) was 3.8-6.6, and the inter-assay CV was 6.7-17.3 %.

##### 5-HydroxyIndole Acetic Acid

5-HIAA was assessed by Bioassay ELISA kit (Shanghai Crystal Day Biotech CO., Ltd, China) based on the Biotin double antibody sandwich technology. The 5-HIAA unit was reported in mmol/l. The intra-assay CV was <10 % and, the inter-assay CV was <12 %.

##### 5-HIAA to 5-HT ratio

Because of the difference in 5-HIAA and 5-HT units, both variables were converted to nmol/l by multiplying them into 10^-6^ and 5.7, respectively, and then the ratio was calculated.

### Statistical analysis

The statistical analysis was performed by SPSS 25 for Windows. For comparison of the baseline characteristics and demographic data between the groups, we used chi-squared test (for qualitative variables) or independent t-test (for quantitative data). Data from the outcomes of the questionnaires (as IBS-SSS, IBS-QoL, HADs and VSI) and blood tests (as 5-HT, 5-HIAA, and 5-HIAA/5-HT) were analyzed by independent t-test or Mann-Whitney U-test for comparison between groups. Moreover, we used paired t-test or Wilcoxon paired rank test for within group comparisons. Normality distribution of data was assessed by Graphical methods, the degree of skewness and normality tests were determined by Kolmogorov–Smirnov and Shapiro-Wilk test. ANCOVA model was used for adjusting variables with significant differences at the baseline. The data are reported as numbers and percentages for qualitative variables, mean ± SD for parametric data and median (25^th^, 75^th^ percentile) for non-parametric data in this article. Statistical significance was considered as P-values <0.05.

## Results

### Enrollment and study completion

**Figure 1:**
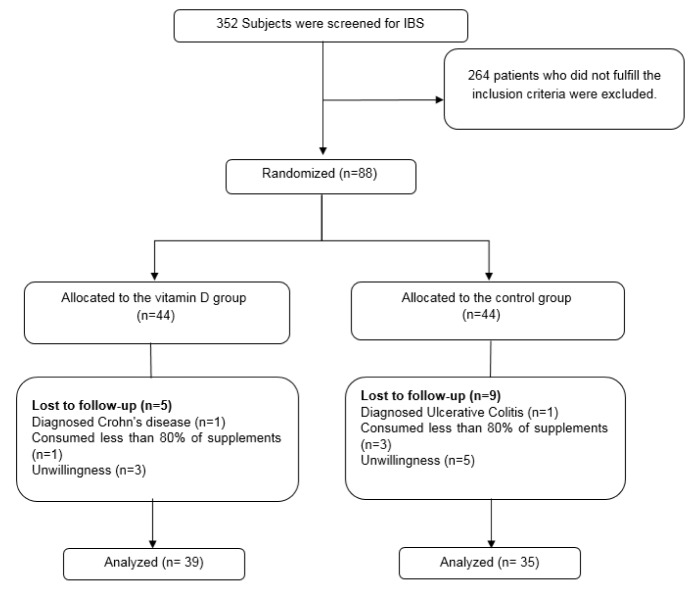
Flowchart of patients from enrollment to the end of the study

A total of the 88 IBS-D patients were included in the study, and 74 of them completed the study. Due to consuming less than 80 % of supplements, diagnosis of inflammatory bowel disease, and unwillingness to cooperate, 14 participants were excluded from the study. The flowchart of the patients enrolled in the study is presented in Figure 1(Fig. 1).

### Characteristics of the study participants

The data of the participants who completed the study (39 women and 35 men, age: 35.51±10.43 years, BMI: 25.32±4.58 kg/m^2^) were included for the analysis. All patients had diarrhea-predominant IBS, based on ROME IV criteria, and moderate disease severity (238.24±50.81) evaluated by the IBS-SSS questionnaire. There was no significant difference between the two groups regarding the baseline characteristics, score of WGO questionnaire, physical activity, and IBS-SSS scores between (Table 1(Tab. 1)). There was no significant difference between two groups in serum 25(OH) vitamin D3 at the beginning of the study. We found no differences in the intake of macronutrient and micronutrient between groups on the basis of the 3-day food records analysis, except for beta-carotene (P=0.04) which we adjusted for possible effect of antioxidant on the main study outcomes (data are not shown).

### Severity of IBS symptoms 

At the end of the study, the total score of IBS-severity score system was significantly improved in both groups compared to the beginning of the study (P<0.001 and P<0.001, respectively). This improvement was higher in the group receiving vitamin D and the difference between the groups was also significant (P<0.001). In subscale analysis, all symptoms ameliorated in both groups except for abdominal pain duration in the control group. Comparing this effect between the two groups, abdominal pain severity, abdominal pain duration, bowel habit satisfaction and life disruption significantly improved in vitamin D group compared to the control group. There was no significant difference in abdominal distention severity between the groups at the end of the study (Table 2(Tab. 2)).

### Quality of life

Overall score of IBS-QoL significantly improved in both groups of vitamin D and placebo at the end of the study (P<0.001 and P=0.007, respectively); however, the vitamin D group showed more improvement than the control group (P=0.049). Moreover, we observed improvement in quality of life subscales in both groups, except for social reaction and relationships in the control group. Dysphoria, interference with activity, health worry, social reaction, and relationships showed more favorable change in the group receiving vitamin D as compared to participants receiving placebo (Table 3(Tab. 3)).

### Anxiety and depression

The results of HADs questionnaire showed that mean anxiety score and mean depression score of patients were 10.47±5.37 and 6.74±4.76, respectively at the baseline of the study. According to the results of the questionnaire, the total score of patients for depression scale was in normal range and for stress was in borderline range. At the end of the study, anxiety decreased significantly in both groups of vitamin D and control compared to the beginning of the study (P=0.004 and P<0.001, respectively); however, this improvement was not significant between the groups. The mean score of depression decreased significantly in vitamin D group (P=0.008), after modifying the effect of confounders; however, the reduction of depression symptoms was still significant between the groups (P=0.023) (Table 4(Tab. 4)).

### Visceral Sensitivity Index

As Table 4(Tab. 4) displays, VSI has improved significantly in both groups of vitamin D and control (P<0.001, P=0.044, respectively). This improvement is significantly greater in the treatment group than in the control group (P=0.008).

### Serum 25(OH) vitamin D3 levels

At the baseline, participants had an insufficient vitamin D level in both groups. After 9 weeks of supplementation with vitamin D, serum 25(OH) D3 significantly increased compared to the baseline values in the treatment group (P<0.001) and reached to normal levels. In addition, there was a significant increase in the vitamin D group compared to the control group at the end of the study (P<0.001) (Table 4(Tab. 4)). 

### Serum Serotonin (5-HT) and 5-Hydroxy-Indole Acetic Acid (5-HIAA)

5-HT significantly was reduced compared to the baseline values in patients receiving vitamin D (P=0.025). Despite this favorable outcome, the effect of vitamin D on serum serotonin, there was no significant statistical difference as compared to the control group at the end of the study. The result of serum samples assessment showed an increase in 5-HIAA in the vitamin D group; however, this rise was not statistically significant. Moreover, there was no significant difference between the two groups at the end of the study. For 5-HIAA/5-HT, same as 5-HIAA, we did not observe any significant change in serum samples of both groups after the intervention compared to the baseline (Table 4(Tab. 4)).

## Discussion

The results of this randomized clinical trial showed that vitamin D3 supplementation improved IBS symptoms, quality of life, depression and visceral sensitivity index as compared to the control group. No significant differences in anxiety, serum concentration of serotonin, 5-HIAA, and 5-HIAA/5-HT were found between vitamin D and control groups. 

The findings of this study showed that intake of one pearl of 50,000 IU vitamin D3 weekly for 9 weeks increased serum 25(OH) vitD3. In line with the findings of our study, it has been reported that nine weeks supplementation with 50,000 IU dose of vitamin D3, does not have any side effects for subjects with vitamin D deficiency (Holick et al., 2011[25]; Roth et al., 2012[56]). Similarly, assessing the effects of vitamin D on IBS, some researchers have shown the same results as our study (Abbasnezhad et al., 2016[1]; El Amrousy et al., 2018[15]; Jalili et al., 2016[27]).

The results of our study and some previous studies show, vitamin D can significantly improve symptoms and quality of life in IBS patients. El Amrousy et al. (2018[15]) evaluated the effect of 2,000 IU vitamin D3 daily in 112 IBS adolescents with vitamin D deficiency for 6 months. The authors reported improvement in serum 25(OH) vitD3, the severity of symptoms and quality of life of patients in the vitamin D group as compared to the control group. Abbasnezhad et al. (2016[1]) supplemented 50,000 IU vitamin D3 biweekly for 6 months in patients with IBS and noticed that taking vitamin D can reduce gastrointestinal symptoms and increase the quality of life of these patients as compared to the placebo-receiving group. In the study of Jalili et al. (2016[27]) on co-administration of soy isoflavones and vitamin D in the management of irritable bowel disease, it was observed that co-administration of soy isoflavones with vitamin D did not improve the IBS-SSS and IBS-QoL; however, prescription of vitamin D alone improved clinical symptoms and quality of life. The exact function of vitamin D in the improvement of clinical symptoms and quality of life of patients with IBS is unclear. It is assumed that vitamin D can control depression, anxiety, and mild inflammation which may be due to genetics, intestinal microbial, inflammation after infection, nutritional factors, etc., thereby improving the clinical symptoms and quality of life of IBS patients (Li et al., 2014[39]; Sinagra et al., 2016[61]). We observed patients receiving placebo significantly improved in symptoms from the baseline. This effect can be due to the psychological problems in IBS patients (Lee et al., 2015[37]). The systematic review of Flik et al. (2017[17]) remarks that the placebo could affect the psychiatric problems of IBS patients because their expectation from and willingness for treatment are more important than the drug. 

Vitamin D and its metabolites have the ability to cross the blood-brain barriers and their receptors are present in different regions of the brain which can contribute to the pathophysiology of depression (Eyles et al., 2005[16]). A systematic review and meta-analysis conducted by Anglin et al. (2013[2]) showed that there was a direct correlation between vitamin D deficiency and adult depression. However, some trial studies have indicated that supplementation with vitamin D cannot affect depression symptoms. It has been reported that the effect is associated with severity of clinical symptoms of depression due to the finding that vitamin D is more effective in people with more severe symptoms (Li et al., 2014[39]). In our study, patients in vitamin D group had a significant reduction in depression symptoms, and the difference between the groups was significant. Some recent studies have shown that there is a relationship between vitamin D levels and anxiety. The animal study of Kalueff et al. (2004[31]) on mice lacking vitamin D receptors in the brain has shown increased stress in these mice compared with the control group. A direct correlation between the severity of anxiety symptoms and low levels of serum vitamin D has also been observed in some human studies (Armstrong et al., 2007[3]; Bičíková et al., 2015[7]). A high prevalence of anxiety and depression in people with IBS has been reported as one of the main causes of this disease. For this reason, the treatment of anxiety and depression in these patients can help to reduce the clinical symptoms (Banerjee et al., 2017[5]). Our results showed that stress score of HADs questionnaire reduced in both groups but the difference was not significant between the groups. The similar borderline range score of anxiety at the baseline led to the absence of a meaningful difference between the two groups at the end of the study; patients did not show severe symptoms of depression and anxiety. It is likely that we might have observed a significant difference between the groups if the duration of the study was longer. Jorde et al. (2008[30]) found that overweight and obese individuals had a significant improvement in symptoms of depression by vitamin D intake of 20,000 and 40,000 IU weekly over a period of one year.

About half of people with IBS report increasing visceral sensitivity (Kanazawa et al., 2011[32]). Psychological disorders such as stress and depression are reported to be the main causes of this sensitivity (Garland et al., 2012[21]). The mechanism of the effect is not clear, but as discussed in some studies on visceral sensitivity in IBS patients, changes in neuromuscular immunity and disorders of intestinal microbial regulation can be the causes of this sensitivity (Cong et al., 2018[9]). There is no specific treatment for this disturbance and the treatment can be in the improvement of clinical symptoms, anxiety, depression, dietary regulation and physical activity of patients (Trinkley and Nahata 2014[66]). Improvement in visceral sensitivity seems reasonable, as our participants had an improvement in clinical symptoms in both groups receiving vitamin D and placebo. On the other hand, we found a better status in the vitamin D group than in the control group, which can be due to the effects of the vitamin on inflammatory and intestinal microbial (Li et al., 2015[40]).

Serotonin is one of the key factors in gastrointestinal movements and secretions the production of which will be increased in people with IBS-D and cause diarrhea and spasms in these patients (Saha 2014[58]; Sen et al., 2011[60]). Any mild inflammation and immune response can lead to hyperplasia of enterochromaffin cells, increased serotonin production and secretion and reduced SERT expression, as a result of which IBS clinical symptoms appear (Thijssen et al., 2016[65]). In addition to increased production, some studies have shown that impaired serotonin metabolism occurs in IBS, thereby decreasing the production of 5-HIAA and its level in the blood and urine levels (Houghton et al., 2003[26]; Jin et al., 2016[28]). A high incidence of vitamin D deficiency in these individuals can worsen inflammatory status (Li et al., 2015[40]; Williams et al., 2018[67]). The study of Dussik et al. (2018[14]) on gene expression profiling and assessment of vitamin D and serotonin pathway variations in patients with irritable bowel syndrome showed that IBS patients’ derived RNA exhibited lower levels of tryptophan hydroxylase-1 (TPH1) expression, the enzyme that catalyzes the rate-limiting step in serotonin biosynthesis in enterochromaffin cells. Also, the level of 25 (OH) vitD3 in patients with IBS (particularly in the IBS-D subtype) was lower than in the non-IBS control. As a result, the expression of selected IBS genetic biomarkers (TPH1) was shown to be modulated by vitamin D. The results of that study suggest that IBS pathogenesis and pathophysiology may be involved in dysregulation of serotonin production and vitamin D insufficiency (Dussik et al., 2018[14]). Moreover, studies on brain monoamine oxidase (Serotonin degrading enzyme to 5-hydroxyindol acetic acid) have shown that treatment with vitamin D can increase the level of expression of this enzyme (Pertile et al., 2016[53]; Sabir et al., 2018[57]). In addition, vitamin D increases the expression of SERT in brain neurons (Sabir et al., 2018[57]). Patrick and Ames's studies on patients with mental disorders have illustrated that vitamin D has a regulatory effect on tryptophan hydrolysis enzymes, indicating that more tryptophan is converted to serotonin in the brain by increasing the activity of the TPH2 enzyme. In non-brain tissues the production of serotonin is reduced and regulated by decreasing the activity of TPH1 (Patrick and Ames, 2015[51], 2014[52]). According to those research findings, and despite the decrease and increase in serum serotonin and 5-HIAA, there was no significant difference between the two groups in our study. Considering a significant improvement in clinical symptoms of patients compared to the control group, there is a strong likelihood that if the duration of the intervention had been longer or biochemical evaluations had been assessed more accurately, we might have seen a significant difference with the control group. 

The strengths of the present study are a first study which has evaluated the effects of vitamin D on anxiety, depression and the biochemical parameters of serotonin and 5-HIAA in patients with IBS. A VSI questionnaire was used to show the status of the patients, and to assess the effect of stress on IBS patients more precisely. This study was performed only in the group with diarrhea-predominant IBS. However, the study has some limitations. Due to time constraints, the duration of our study was 3 months. Lack of evaluation of 5-HT and 5-HIAA in platelet poor plasma, which according to other studies is the best method to measure these factors, is another limitation of our study (Bearcroft et al., 1998[6]). This method requires the employment of a higher number of kits; however, due to budget limitation, we could not carry out this method.

## Conclusion

The findings of the present study revealed that supplementation with vitamin D3 in patients with diarrhea-predominant irritable bowel syndrome could improve severity of symptoms, quality of life, depression and visceral sensitivity. The findings also showed that intake of vitamin D reduced anxiety and serum serotonin; however, this decrease was not significant compared to the control group. 

We recommend that serum 25(OH) vitamin D3 should be evaluated in the treatment process of these patients. Further studies are needed to confirm the effect of vitamin D on IBS patients and determine the exact mechanism.

## Acknowledgements

We are owed to the IBS patients for participating in this study; and for their collaboration in the implementation and completion of this project. We are so grateful for the cooperation of Dr. Nasseri Moghadam, and Dr. Merat (Tehran University of Medical Sciences) to accomplish this project.

## Funding

This research did not receive any specific grant from funding agencies in the public, commercial, or not-for-profit sectors.

## Authors’ contributions

The authors' responsibilities were as follows: MKS and FS designed the research; MKS, MM, MM, AHFK, SA and NA contributed to sampling; MKS and LJ analyzed and interpreted the data; MKS and FS wrote the original draft; MKS, AD, FS and SS contributed to reviewing and revising the paper. All authors read and approved the final manuscript.

## Declaration of competing interests

The authors declare that there were no conflicts of interest.

## Figures and Tables

**Table 1:**
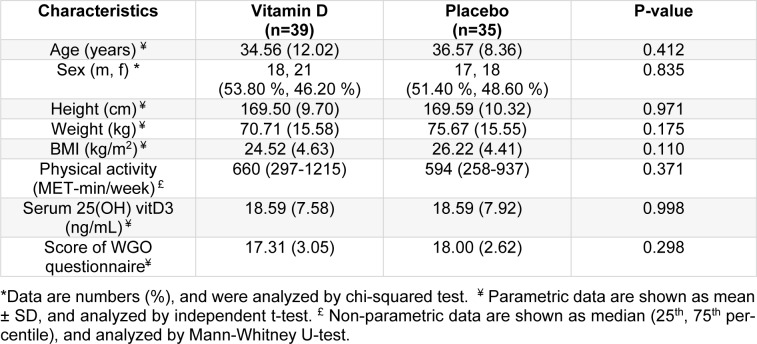
Baseline characteristic of study participants

**Table 2:**
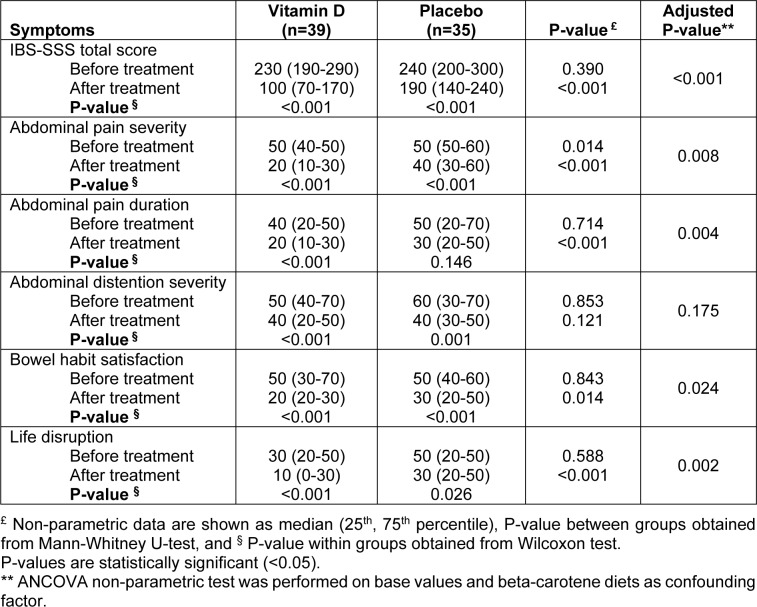
Comparisons of severity of symptoms before and after intervention in vitamin D and placebo group in patients with irritable bowel syndrome

**Table 3:**
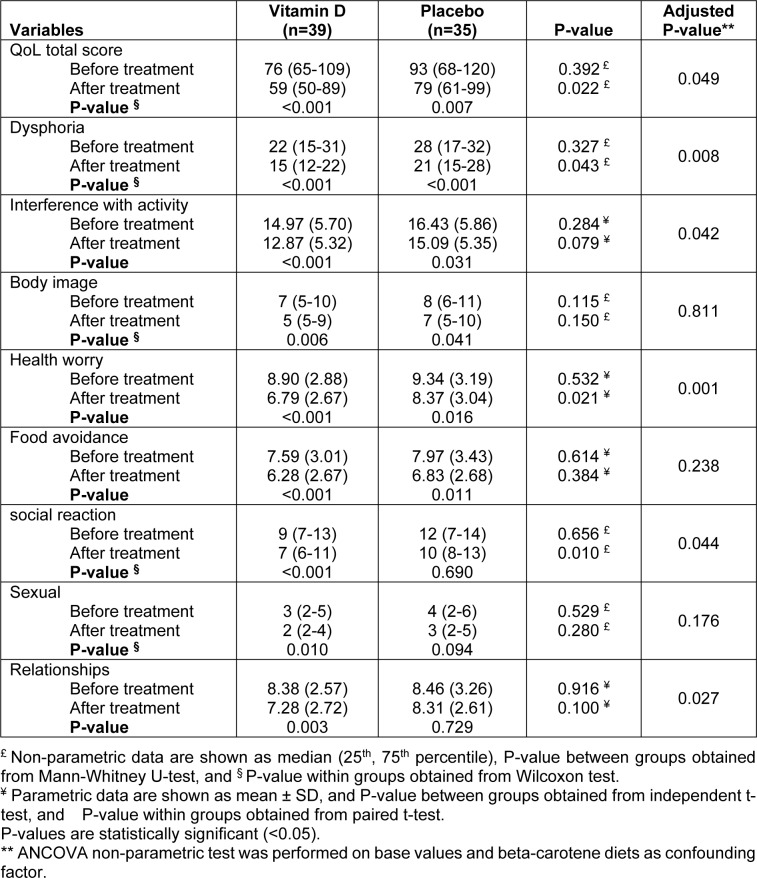
Comparisons of quality of life before and after intervention in vitamin D and placebo group in patients with irritable bowel syndrome

**Table 4:**
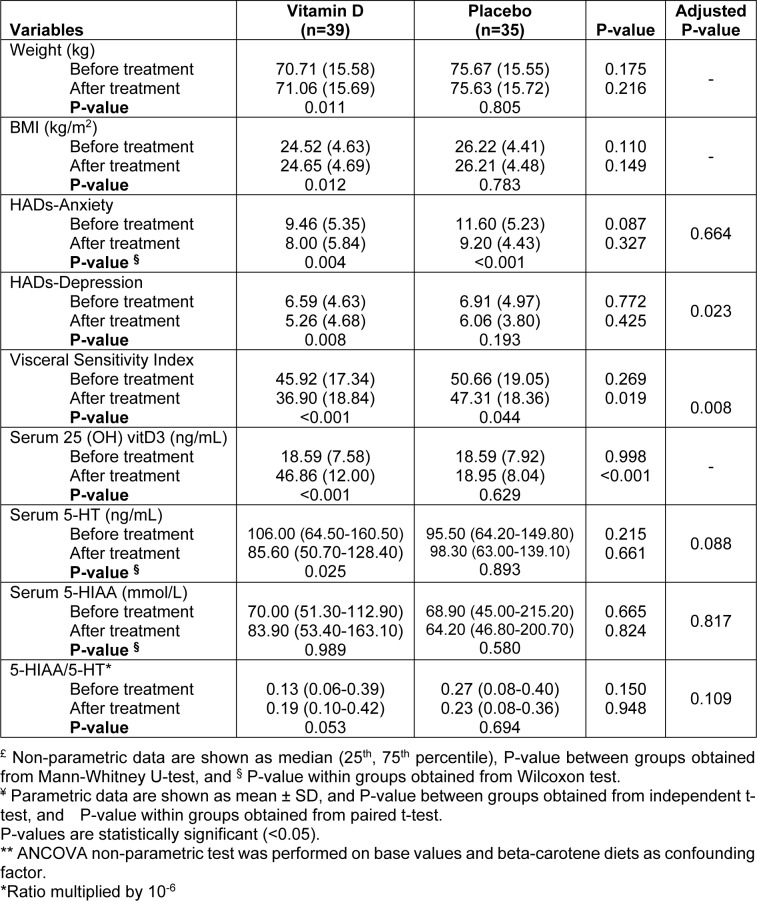
Comparisons of weight, BMI, HADs-Anxiety, HADs-depression, Visceral Sensitivity Index, serum 25 (OH) vitD3, 5-HT, 5-HIAA and 5-HIAA/5-HT ratio before and after intervention in vitamin D and placebo group in patients with irritable bowel syndrome
